# Feasibility of a patient decision aid regarding disclosure of personal health information: qualitative evaluation of the Health Care Information Directive

**DOI:** 10.1186/1472-6947-4-13

**Published:** 2004-09-10

**Authors:** C Shawn Tracy, Guilherme Coelho Dantas, Ross EG Upshur

**Affiliations:** 1 Primary Care Research Unit, Sunnybrook and Women's College Health Sciences Centre 2075 Bayview Avenue, Room E3-49 Toronto, ON M4N 3M5 Canada; 2 Department of Family and Community Medicine, University of Toronto 257 McCaul Street, 2nd Floor Toronto, ON M5T 2W5 Canada; 3 Department of Public Health Sciences, University of Toronto McMurrich Building, 12 Queen's Park Crescent West Toronto, ON M5S 1A8 Canada

## Abstract

**Background:**

Concerns regarding the privacy of health information are escalating owing both to the growing use of information technology to store and exchange data and to the increasing demand on the part of patients to control the use of their medical records. The objective of this study was to evaluate the Health Care Information Directive (HCID), a recently-developed patient decision aid that aims to delineate the level of health information an individual is willing to share.

**Methods:**

We convened a series of four focus group meetings with several communities in a large Canadian city. A total of 28 men and women participated, representing health care consumer advocates, urban professionals, senior citizens, and immigrants who speak English as a second language. Data were analysed using qualitative methods.

**Results:**

Participants lacked substantial knowledge regarding the fate and uses of personal health information. They expressed mistrust concerning how their information will be used and protected. Several suggestions were made towards customizing the use of data according to specific needs rather than broad and full access to their charts. Furthermore, despite concern regarding the implementation of a tool like the HCID, participants were hopeful that a refined instrument could contribute to the improved regulation of health information.

**Conclusion:**

This study indicated poor knowledge concerning the uses of personal health information, distrust concerning security provisions, and cautious support for a patient decision aid such as the HCID to improve control over health data.

## Background

Health information privacy has come to the forefront of ethical concern in the early 21^st ^century [1]. The advent of electronic health records, information technology, and large databases (such as administrative and genetic) with the potential for extensive linkages have raised concerns about the security of health information [2,3]. The details of where such information flows, who has access, and for what purposes have assumed paramount importance [4-6].

Health information is valuable for numerous purposes, first and foremost for patient care, but also for secondary uses including hospital administration and health services research [7,8]. Such non-clinical care uses are required for system performance and evaluation, as well as to answer questions about disease trends and health outcomes. In general, there is a *prima facie *obligation for the protection of such intimate and privileged information [4,8]. Scientific surveys and public opinion polls have shown that access to medical records is generally considered appropriate after consent has been obtained for a specific use [9-12]. Yet, this obligation admits to several exceptions and there is lack of clarity as to whether express consent is required for each and every use. Some have argued that requiring unique individual informed consent for each use of health information would be burdensome [13-15]; however, there are public opinion data suggesting that legislative initiatives to require such consent would be viewed favourably [9].

Internationally, legal initiatives have proposed solutions to the dilemma posed by health information. In Europe and North America, such initiatives have led to restrictive legislation that, according to some commentators, may endanger public health goods [4,16,17]. A privacy paradox thwarting further progress has been identified: individuals want both the guaranteed privacy of their personal health information (PHI) and the public benefits that accrue from the use of medical records [18]. Two distinct avenues have been proposed for the solution of this paradox; the first avenue concentrates on governance issues, whereas the second promotes the development of tools, programs, and systems to enhance the lay understanding of and control over the uses of health data and thereby facilitate informed consent for secondary uses, as advocated by Mandl and his colleagues [19]. This paper pursues the second avenue.

In 2001, Upshur and Goel first proposed the Health Care Information Directive (HCID), a patient decision aid analogous to an advance directive in end-of-life care [20]. The underlying logic was to combine ethical appropriateness of use of PHI with the sensitivity of the data. As shown in Table 1, the HCID clearly presents the various permutations and combinations of sensitivity and usage in the form of a matrix. The goal of the tool is to allow individuals to make informed choices about the specific types of health information they are willing to disclose (if any) for a number of specified purposes.

In the original proposal, it was stated that pilot testing of the tool, and revision on the basis of such testing, is required [20]. In order to assess the feasibility of implementing the HCID, two segments of the population were studied: the general lay public and privacy experts such as ethicists, academics, and provincial privacy commissioners. In this paper, we report the results of the study conducted with the general public. Specifically, our objective was to investigate lay knowledge of the uses and accesses of health information and to solicit feedback on the prototype of the HCID.

## Methods

### Participants and setting

The study was set in Toronto, Canada, a large, culturally-diverse urban centre. In order to sample multiple views and perspectives, a series of focus group meetings was convened with the following four groups: senior citizens, urban professionals, immigrants with English as a second language, and consumer advocates. The latter group was comprised of volunteer members of a well-known national consumer advocacy association for which health care is an issue of primary interest and activity. We opted to employ focus group methodology as opposed to individual interviews or a questionnaire survey in order to capitalize on the effects of group interaction. These particular communities or target groups were selected in order to maximize the variability within our sample on important demographic characteristics such as age, gender, occupation, education, and native language, etc. (available funding allowed for a total of four focus groups). Potential participants were recruited using various methods: the seniors were recruited through a local community centre offering programs for senior citizens; participants in the immigrant/ESL group were recruited through an immigrants group at another community centre; the urban professionals were recruited by way of posters and fliers distributed at a number of hospitals and university sites; and the advocates were recruited through a national consumer advocacy association. In all cases, initial contact with potential participants was made either by telephone or e-mail and then followed up with formal letters of invitation describing the project. The focus group meetings were held in a convenient location for the participants and lasted for up to two hours.

We developed a topic guide for the focus group meetings in order to address the main issues related to the feasibility of the HCID (see Appendix 1). The topic guide was developed according to the principles of formative evaluation (also known as 'developmental' evaluation) [21]. Focus group participants were initially questioned regarding their understanding of health information and its uses. Participants were then introduced to the HCID, which was presented as a patient decision aid currently under development by "university researchers." Participants were informed that their feedback and suggestions would guide the continuing refinement of the tool and were asked to be as candid as possible. Following a brief period of approximately 5–10 minutes in which participants examined the HCID and jotted down any questions/concerns/suggestions, participants were asked to share their thoughts on its relative strengths and limitations. As per the topic guide, issues of content, utility, and feasibility were addressed in turn, followed by a discussion of perceived benefits and burdens. At the end of each meeting, participants were provided an opportunity to raise any issues or concerns that had not been previously addressed.

Two of the authors (GCD and CST) moderated the four focus group meetings, which lasted 90 minutes on average. A total of 28 participants took part (see Table 1 for a description of the sample). The meetings were audio-recorded with participants' consent and transcribed verbatim by a professional transcriptionist. To ensure accuracy and to clarify any muffled passages, all transcripts were verified by one of the two group moderators. Participants received an honorarium of $50 for their time, in addition to transportation costs.

The study was approved by the Research Ethics Board at Sunnybrook and Women's College Health Sciences Centre and the Office of Research Services at the University of Toronto. All participants signed and returned a consent form.

### Data analysis

The analytic process was one of thematic content analysis. The topic guide developed for the focus group meetings served as the basis for the data analysis process. Two of the authors (GCD and CST) independently read each of the transcripts and identified passages of text relating to each of the various key issues from the topic guide (e.g., content, utility, benefits, etc.) which, for the purposes of coding and analysis, served as the macro-codes. Following this step, lists of themes were constructed and then compared. The transcripts were then independently coded according to an agreed-upon coding scheme. Tests for inter-coder reliability indicated a high level of agreement among the two coders; instances of disagreement were resolved through a process of discussion and negotiation. To strengthen the validity of the findings, the analytic processes of coding and interpretation were reviewed by the senior author (REGU). The results of our analysis are reported according to five key themes: participant knowledge and understanding of health information and its uses; control of access to health data; mistrust of data security provisions; need and utility for a tool such as the HCID; and, finally, perceived implementation barriers. Consenus statements reported below are not a reflection of any explicit consensus development techniques, but rather are summary statements of the research team's observation and interpretation of the focus group discussions. For each quotation, a specific code is provided to identify the speaker as a participant in one of the four focus groups (CA = consumer advocate; IM = immigrant; SC = senior citizen; and UP = urban professional).

## Results

### Knowledge and understanding

The majority of participants possessed extremely limited knowledge of how their PHI is collected, used, and disclosed. Many confessed to having given little or no thought to the issues involved in the health privacy debate. This was particularly true for recent immigrants: "I think the truth is that I don't know. I've never thought of that before, who has my information."(IM-1). The level of understanding was low among participants in other groups as well, as a number of comments betrayed basic misperceptions of how PHI is currently managed within the Canadian health care system:

"It [personal health information] goes in the computer and then it's available to every medical professional in Ontario." (SC-3)

"Health providers have access to your file, to your information... but everybody in the financial department, too, because they have to bill OHIP [Ontario Health Insurance Program] so they have to know everything about you." (IM-2)

One participant perceptively noted that the general population has limited knowledge of the issue of health privacy:

"That's another thing. What do people know about what they can get access to, what they can ask for, and what they can expect? I think the majority of the population have no idea of what they can ask for and expect to get." (CA-4)

### Control of access

Participants' accounts clearly suggested an absence of patient control over the collection, use, and disclosure of PHI. No participants recalled having ever been consulted about how their information was to be used. A great deal of concern was voiced about the extent to which health data appears to be freely accessible to a wide variety of users: "Lawyers, psychologists, social workers, researchers, pharmaceutical companies. Where does it stop?" (UP-4). In the course of describing how they feel about the issue of health privacy, participants repeatedly used terms such as "scary" and "horrifying":

"I'm scared to guess who has [access to my health information]. It looks so easy for a lot of people to have access. That's the scary part of it. Maybe your employer can have access to your files, too. I don't know, that's just a guess." (IM-6)

The majority of participants expressed concern that their PHI is not adequately safe-guarded and that the implementation of a tool such as the Health Care Information Directive would not result in significantly enhanced privacy or increased security. There was a widespread view that too much data is currently made available when only specific details are required. Doubts were raised about how consent for one specific use only would be managed:

"What's going to prevent any leaking from one of these [uses] into the others? ... It just seems to me that if there's information on-line, things are going to be compromised. You know, people make a living doing that stuff. The more they find out about you, the more you can be exploited. It's that simple. These systems, they're not secure yet, and I don't know if they can ever be secure." (UP-3)

Health privacy concerns related to the security of electronic databases and the Internet were shared by others:

"We've all heard stories where there's been stolen identities. How difficult is it for the victim to get his or her own identity back? Same idea. Where does it end? Where does it stop? Who's got what information? How am I going to protect myself?" (UP-6)

Participants suggested a number of other mechanisms that could work in conjunction with the HCID to enhance security and facilitate individual control over PHI. One such mechanism would be an online real-time audit system in which the details of all accesses to an individual's PHI are recorded and made available to those wishing to track access to their PHI over time. Also, the idea of a health data ombud was raised in several groups and received a great deal of support.

### Mistrust

Issues related to trust were raised in each of the four groups. Participants of all ages and socioeconomic status expressed feelings of mistrust in relation to the protection of their privacy and the security of their PHI. A great many participants spoke of how their past experiences with the health care system have fostered significant mistrust and suspicion where their right to privacy is concerned. These accounts revealed a growing distress that large corporations have too much access to and influence on government programs, especially in contrast to the access and influence accorded to patients:

"What about the rights of the patient? Let's say I'm the patient. What kind of power do I have? Let's say this [the HCID] was created next year. What power does the patient have to make sure any of this is happening? To me, a pharmaceutical company is way more powerful than the patient." (CA-2)

Others were even more sceptical, questioning the trustworthiness of the basic tenets of the model upon which the HCID is based:

"By filling this out, I'm buying into the concept of sharing information, but I don't have any faith that it can be kept private.... It will spill, it will bleed, it will flow. So I'm distrustful of the whole thing. This just sets up more spilling and more flowing. If I fill out a form like this, then I'm validating the process, which I don't really trust." (UP-8)

### Need and utility

While the majority were sceptical that the HCID would prevent all breaches of privacy, there was a general consensus that it would serve to enhance significantly the security of health data. One participant noted that the proposed decision aid may also serve a useful purpose as "a sort of consciousness raising" tool. Other impressions varied from "it has some potential" to "it is a great step forward." Reactions were mixed in response to the question of whether the HCID will be successful in empowering individuals and increasing the amount of control over PHI:

"I'm very dubious as to whether this matrix will be useful because of the difficulty people will have filling it out. In spite of that, I think the idea has merit and principle. There's merit in what you're trying to do, but I don't think that this is going to succeed." (CA-5)

"I guess the reality is our information will be shared, so we might as well get on the bandwagon with regulating it and controlling it... You can't stop it from being shared, so maybe you can influence how it will be shared." (UP-2)

Despite the weaknesses and limitations of the present version of the directive, one participant neatly summarized the view of the majority of participants regarding the utility of the HCID or some such tool: "Not having it allows total absence of control, therefore it is a necessary evil." (CA-4).

### Implementation barriers

Participants provided numerous suggestions regarding the formatting of the HCID in order to facilitate implementation. Ideas ranged from simplifying the language and providing definitions of technical terms to modifying the layout and shading those areas where there is no discretion (i.e., for physician payment):

"Maybe you've got too many columns.... Well, maybe you're trying to do too many different things at once." (CA-4)

"This is too busy, it's too much. If I'm sick, I friggin' don't want to be bothered with it.... Look at this. English is my first language. How would somebody whose mother tongue is something other than English? It's too complicated." (SC-1)

"I think people tend to say 'no' for things that are not clear. I would say 'yes' if I knew what it means exactly, but I don't know, so I don't want to take a chance." (IM-1)

To address the complexity issue, suggestions were made concerning the need to provide a customer service representative either in a health clinic or via a toll-free helpline for assistance with completing the HCID.

Across the four groups, there was great variability in the preferred mode of implementation. The preference among participants in the health advocates and urban professional groups was for an on-line implementation format. In contrast, the majority of the senior citizens and immigrants preferred other options, the former favouring a postal format and the latter the primary care setting. As one senior citizen remarked: "I prefer the doctor's office. I wouldn't fill it and send it back through the mail, no." (SC-3)

## Discussion

A recent editorial in the *British Medical Journal *suggested that perhaps patients should be asked whether certain items of their medical chart should only be shared with specified individuals or organizations or only for pre-determined purposes [16]. This paper reports the evaluation of the feasibility of a tool that seeks to accomplish exactly that purpose, namely, greater patient control over how personal health information is used and disclosed.

Study participants lacked substantial knowledge regarding the fate and uses of PHI within a publicly-funded health care system. Participants expressed mistrust concerning how their PHI is used and safe-guarded. Several suggestions were made towards customizing the use of data according to specific needs rather than broad and full access to their charts. Furthermore, concerns were expressed regarding the implementation of a tool such as the HCID. Nevertheless, there was hope that a refined instrument could contribute to improved data management and regulation and enhanced privacy protection.

Although this study reports on a small sample from a single large urban centre, the focus group participants were drawn from various different niches of Canadian society. This sampling strategy allowed us to explore a broad range of experiences and perspectives; however, further testing and evaluation are required. Ultimately, it will be necessary to evaluate the tool using a representative sample of patients who complete the HCID in a 'live' test of its feasibility.

Our findings underscore the difficulties involved in accessing health care data for research and other secondary purposes. Participants acknowledge the myriad benefits derived from the use of health data; however, distrust, lack of respect, and insufficient patient control of the process threaten to undermine these very benefits. This finding has been previously reported by Willison and associates in Canada [22] and by Robling and colleagues in the UK [12]. The present results also suggest that the education and information needs of diverse groups such as seniors and immigrants who speak English as a second language should be taken into account when considering strategies to enhance individual control over PHI and minimize the problem of authorization bias when utilizing health information for secondary purposes.

Participants appreciated the benefits accorded by a tool such as the HCID. As opposed to forms of blanket consent or other opt in/opt out models, the possibility of exerting greater control over one's PHI was attractive. The participants provided concrete suggestions for improving the format and content of the HCID. It is evident that any method to enhance control of health information via explicit consent requires description of the various forms the data may take, the specific purposes for which the data would be used, and the various channels of the health care system through which the data might flow. To our knowledge, such detailed data flowmaps for PHI do not exist in Canada, although they have been laid out in Great Britain [23]. A model has been proposed by Schoenberg and Safran [24]. The creation of such maps is of high priority.

An intriguing finding was the appeal of an online data audit system. Possessing the ability to monitor who has accessed their PHI and for what purposes raises the possibility of additional strategies that could empower individuals to control the fate of their health information. This finding has also been verified by Pyper and colleagues [25] and was previously highlighted by MacDonald [26]. As well, the concept of a data ombudsperson was considered attractive to a number of participants, indicating that an improved governance framework would be acceptable to some segments of the population.

Finally, there is a distinction to be made between using the HCID to enforce the will of the patient versus its use as a documentation tool. Our vision is that the HCID will, ultimately, serve both of these important functions; further follow-up evaluation of a revised model of the HCID using a larger sample (comprised of patients as well as providers) is needed to address this distinction. As with any patient decision aid or empowerment tool, documenting the preference of the patient is only meaningful and useful to the extent that the documented preferences are known and ultimately acted upon by those providing care. We believe the present data illustrate the critical problem of mistrust that currently exists. Indeed, this is one of the greatest challenges to be overcome in the continuing development and validation of this tool.

## Conclusion

This study indicated poor knowledge concerning the uses of health data, distrust concerning current security provisions, and qualified support for a tool such as the HCID to improve patient control over health information. On the basis of this evaluation, the HCID will be revised significantly, including the addition of an educational component, and then submitted to further evaluation. The creation of data flowmaps and the exploration of audit functions and governance structures are strongly recommended as avenues for future research.

## Competing interests

None declared.

## Authors' contributions

CST participated in the collection, analysis, and interpretation of the data and is the primary author of the paper. GCD recruited the participants, moderated the focus group meetings, participated in the data analysis, and contributed to the editing and revising of the paper. REGU initiated and designed the study, participated as a reliability check in the process of data analysis, and contributed to the editing and revising of the paper. As principal investigator, he will act as guarantor. All authors have read and approved the final version of the paper.

## Appendix 1

### FOCUS GROUP TOPIC GUIDE: EVALUATION OF THE 'HEALTH CARE INFORMATION DIRECTIVE'

#### A. Personal Health Information

1. What is your understanding of the term 'personal health information'?

2. Who do you believe has access to your personal health information?

3. Do you believe that consent should be required to access your personal health information?

#### B. Content

1. What is your first impression of the Health Care Information Directive?

2. Is it clear?

3. Is it self-explanatory?

#### C. Utility

1. How useful do you believe the Health Care Information Directive would be in practice?

2. Is it user-friendly?

3. What kind of changes would you suggest?

#### D. Feasibility

1. How feasible is the application of the Health Care Information Directive?

2. Who should present it to the patient?

3. When should it be presented to the patient?

#### E. Benefits and Burdens

1. What are the potential gains of the Health Care Information Directive?

2. What are the potential harms?

3. Does it adequately protect privacy and confidentiality?

#### F. Additional Comments

## Pre-publication history

The pre-publication history for this paper can be accessed here:



## Figures and Tables

**Table 1:** Demographic characteristics of focus group participants

**Focus Group**	**Number of** **Participants**	**Male:Female** ** Ratio**	**Modal Age** ** Group**	**Some Post-Secondary** ** Education**
Urban professionals	8	4:4	30–39 yrs	8
Immigrants (ESL)	7	2:5	40–49 yrs	5
Health advocates	6	2:4	50–59 yrs	6
Senior citizens	7	0:7	>70 yrs	1
